# The effect of social media and infodemic on mental health during the COVID-19 pandemic: results from the COMET multicentric trial

**DOI:** 10.3389/fpsyt.2023.1226414

**Published:** 2023-07-27

**Authors:** Gaia Sampogna, Matteo Di Vincenzo, Mario Luciano, Bianca Della Rocca, Umberto Albert, Claudia Carmassi, Giuseppe Carrà, Francesca Cirulli, Bernardo Dell’Osso, Maria Giulia Nanni, Maurizio Pompili, Gabriele Sani, Alfonso Tortorella, Umberto Volpe, Andrea Fiorillo

**Affiliations:** ^1^Department of Psychiatry, University of Campania “L. Vanvitelli”, Naples, Italy; ^2^Department of Medicine, Surgery and Health Sciences, University of Trieste, Trieste, Italy; ^3^Department of Mental Health, Azienda Sanitaria Universitaria Giuliano Isontina—ASUGI, Trieste, Italy; ^4^Department of Clinical and Experimental Medicine, University of Pisa, Pisa, Italy; ^5^Department of Medicine and Surgery, University of Milan Bicocca, Milan, Italy; ^6^Center for Behavioral Sciences and Mental Health, National Institute of Health, Rome, Italy; ^7^Neuroscience Research Center, Department of Biomedical and Clinical Sciences and Aldo Ravelli Center for Neurotechnology and Brain Therapeutic, University of Milan, Milano, Italy; ^8^Department of Psychiatry and Behavioural Sciences, Stanford University, Stanford, CA, United States; ^9^Department of Neurosciences and Rehabilitation, Institute of Psychiatry, University of Ferrara, Ferrara, Italy; ^10^Department of Neurosciences, Mental Health and Sensory Organs, Faculty of Medicine and Psychology, Sapienza University of Rome, Rome, Italy; ^11^Department of Neuroscience, Section of Psychiatry, University Cattolica del Sacro Cuore, Rome, Italy; ^12^Department of Neuroscience, Sensory Organs and Thorax, Fondazione Policlinico A. Gemelli IRCCS, Rome, Italy; ^13^Department of Psychiatry, Fondazione Policlinico A. Gemelli IRCCS, Rome, Italy; ^14^Department of Psychiatry, University of Perugia, Perugia, Italy; ^15^Clinical Psychiatry Unit, Department of Clinical Neurosciences, Università Politecnica delle Marche, Ancona, Italy

**Keywords:** pandemic, mental health, social media usage, depression, stress, trauma-related symptoms, infodemic

## Abstract

On January 30, 2020, the World Health Organization (WHO) declared the status of pandemic due to the COVID-19 infection. The initial phases of the pandemic were characterized by uncertainty and public fears. In order to cope with such unexpected conditions, people adopted different coping strategies, including search for information, accessing Internet, and using social media. The present study based on the COMET collaborative research network aims to: (1) assess use of Internet and of social media among the Italian general population; (2) explore differences in web usage between people with pre-existing mental disorders and the general population; (3) identify changes over time in social media usage along the phase 1 of the pandemic; (4) identify the clinical, socio-demographic and contextual predictors of excessive use of social media. A significant increase in time spent on Internet, with an average time of 4.8  ±  0.02 h per day, was found in the global sample of 20,720 participants. Compared with the general population, Internet use was significantly higher in people with pre-existing mental disorders (5.2  ± 0.1 h vs. 4.9  ±  0.02; *p* < 0.005). According to the multivariate logistic regression model, the risk of excessive use of social media and Internet was significantly higher in people with moderate levels of depressive symptoms (OR: 1.26, CI 95%: 0.99 to 1.59, *p* < 0.0.005); while protective factors were being students (OR: 0.72, CI 95%: 0.53 to 0.96, *p* < 0.0029) and living in central Italy (OR: 0.46, CI 95%: 0.23 to 0.90, *p* < 0.002). The evaluation of social media and Internet use by the general population represents a first step for developing specific protective and supportive interventions for the general population, including practical suggestions on how to safely use Internet and social media.

## Background

On January 30, 2020, the World Health Organization (WHO) declared the status of pandemic due to the COVID-19 infection. The pandemic represented a new form of trauma, which was completely unexpected and associated with a high rate of mortality worldwide (1, 2).

During the initial phase of the COVID-19 pandemic, negative effects on the mental health of the general population have been reported (3, 4), as a consequence of the strict lockdown policies, the lack of social interactions and worries about the future. Furthermore, the impact of the COVID-19 pandemic was even higher on at-risk groups (5), including those affected from mental disorders or physical disorders, who had serious difficulties in accessing health services and in many cases experienced a worsening of clinical symptoms (6, 7). It is likely that the long-term consequences of the COVID-19 pandemic on mental health will be even worse in the future given the high tropism of the virus on brain cells (8–10).

Due to the strict containment measures issued for limiting the disorder, levels of anxiety and depressive symptoms, loneliness (11) as well as intimate partner violence and family conflicts (12) increased significantly in the general population. The initial phases of the pandemic were characterized by uncertainty and fears about the future. In order to cope with such unexpected conditions, people endorsed different coping strategies, including denial and joking, or practical coping strategies, associated with the adopting of protective measures (13).

Different individual, social and cultural factors, including coping strategies, cognitive styles, personality traits, socio-economic status and type of governmental containment measures, might have mitigated the impact of the COVID-19 pandemic on mental health. These factors played a crucial role in the process of pandemic adaptation, defined as the capacity to survive in a particular condition (14). Johnson et al. (15) found that people who coped better with the pandemic reported high levels of physical activity, a positive post-traumatic growth and an increase in the quality and quantity of their social network (16).

During the pandemic, many people adopted seeking for information (especially on Internet) as a coping strategy to reduce– or at least manage–anxiety, uncertainty and fear (17). The WHO has defined as excessive the quantity of information available about the pandemic, a phenomenon called “infodemic” (18). During lockdown periods, general population have reported more time than usual on seeking for information through the Internet. From January to May 2020, the term “COVID-19” has been quoted almost 700 million times in digital and social media messages worldwide (19). Many difficulties in separating true and reliable information on COVID-19 pandemic from false and inappropriate information were reported (20). In many cases, such complex and controversial situation has been worsened by conflicting declarations released by government bodies and public health organizations (e.g., the CDC and WHO), nurturing social mistrust and complicating individuals’ decision-making (21). In the current digital era, the excessive usage of Internet and social media, defined as “websites and applications that enable users to create and share content or to participate in social networking” (22), has represented one of the most relevant concerns for general population’s mental health (23–25).

During the different phases of the pandemic, the impact of social media and internet use on mental health has been explored (26). In 2020, 4.54 billion people were using Internet and 3.80 billions were using social media, with an increase of almost 300 millions of users compared to the previous calendar year. Internet and social media have transformed communication styles and information sharing, becoming an essential part of our daily life. According to the International Telecommunication Union (ITU), during the first year of the pandemic, the number of Internet users grew by 10.2%, which has represented the largest increase in a decade. The growth has been less in 2021, with an increase of 5.8%, in line with pre-crisis data.

Several studies found that social media acted as an adaptive coping strategy, reducing the levels of stress and anxiety (27); on the contrary, other studies found increased levels of anxiety in the general population due to overwhelming incorrect and negative information about COVID-19 pandemic found on the web (28, 29). Most of these trials have been carried out in China and in the US (30–33), while only a few investigations have explored this phenomenon in European countries (34, 35).

Italy has been one of the first European countries severely hit by the pandemic, with strict stay-at-home orders and lockdown procedures issued by the Italian government from March 8 to May 3, 2020—defined as “Phase 1” of the public health emergency. Sixty million inhabitants were affected by these containment measures (36). During phase 1, only essential activities were allowed. In this scenario, the usage of social media, Internet and mass-media has represented an essential tool for facilitating at-a-distance communication among people. However, the excessive searching for information might have had a detrimental impact on mental health, in particular when considering specific at high-risk population, such as people with pre-existing mental disorders.

The present study, based on data collected in Italy during the phase 1 of the COVID-19 pandemic within the COMET collaborative network, aims to: (1) assess the use of Internet and social media in Italian general population; (2) explore differences in web usage between people with pre-existing mental disorders compared to the general population; (3) identify changes over time in social media use during the phase 1 of the pandemic; (4) identify clinical, socio-demographic and contextual predictors of excessive usage of social media.

## Materials and methods

### Study sample

The COMET trial is a not-funded study promoted by nine Italian University sites in collaboration with the Italian National Institute of Health, which developed an online survey targeting the Italian adult general population during the first wave of the COVID-19 pandemic in Italy.

The survey was opened for data collection from March 30 to May 2, 2020. The survey was implemented on the EUSurvey web platform, hosted by the European Commission. The survey took from 15 up to 45 min to be completed.

The promotion and dissemination of the survey included a multi-step procedure with direct email invitation to healthcare professionals and to mailing lists of Italian national psychiatric associations, users and carers’ associations; and promotion on social media channels, including Facebook, Twitter, and Instagram.

Only adult population (aged >18 years) were invited to participate, representing the main inclusion criteria. A snowball sampling procedure has been adopted for obtaining a large sample of the Italian population and to evaluate the impact of the studied variables on the outcome measures. The complete study protocol is available in Fiorillo et al. (3).

The primary aim of the current paper was to assess time spent on Internet and social media by the Italian general population and by people with pre-existing mental disorders.

The Ethical Review Board of the University of Campania “L. Vanvitelli” has revised and approved the present study with the following protocol number: 0007593/i.

### Assessment tools

Participants’ socio-demographic and clinical data included gender, age, geographical region, occupational condition, marital status, educational level, living condition, satisfaction about living condition and economic condition; participants’ clinical characteristics included the presence of a pre-existing physical or mental disorder, COVID-19 infection, use of medications for any physical and/or mental disorder.

Time spent on Internet was evaluated through an *ad-hoc* schedule, including items on the main purposes for using Internet and social media. Each item was evaluated on a five-point Likert scale, ranging from 0 = never to 4 = always. The assessment instrument is available as Supplementary material (please see Supplementary material 1).

Other validated and reliable assessment tools used in the survey included: the DASS scale for the evaluation of Depressive, Anxiety and Stress-related Symptoms (37); the Severity-of-Acute-Stress-Symptoms-adult scale (SASS) (38) and the Impact of Event Scale (IES) for trauma-related symptoms (39); the General Health Questionnaire (GHQ) for the evaluation of general wellbeing (40); the obsessive-compulsive inventory for obsessive-compulsive symptoms (41); the Insomnia Severity Index (ISI) for sleep disorders (42); the UCLA loneliness scale for perceived loneliness (43); the Suicidal Ideation Attributes Scale (SIDAS) for suicidal ideation (44); the Brief-COPE scale for assessing type of coping strategies adopted (45); the PTG-inventory for the assessment of levels of post-traumatic growth (46); the Connor–Davidson resilience scale (CD-RISC) for the assessment of the levels of resilience (47) and the Multidimensional Scale of Perceived Social Support (MSPPS) scale for evaluating the quality of social network (48).

### Statistical analysis

Socio-demographic and clinical characteristics of the global sample were analyzed using descriptive statistics, such as mean and standard deviation as well as frequency tables, as appropriate.

In order to identify clinical, socio-demographic and contextual predictors of social media usage, a multivariate logistic regression model has been implemented. The questions included in the survey “How frequently do you use Internet for …?,” have been collapsed in a single variable and then dichotomized in “excessive use” vs. “normal use” of social media/Internet. Based on the available literature, the global score has been transformed in a binary variable using the mean score of 3 as threshold (i.e., >3 “excessive use” was transformed in to “1,” score < 3 was converted in to “0”).

Several confounding variables, such as being infected by COVID-19, having a pre-existing mental disorder, being a healthcare professional, living in specific geographic areas, have been included in the model. Moreover, analyses have been controlled for age, gender, coping strategies, perceived loneliness, levels of general health, presence of insomnia symptoms, as well as for rates of COVID-19 cases and COVID-related mortality rate.

In order to adjust for the probability of participants of being exposed to COVID-19 infection in each week of Phase 1 lockdown, a propensity score was used. This statistical approach was selected since it produces a better adjustment for differences at baseline, rather than simply including potential confounders in the multivariable models. The propensity score was calculated using as independent variables age, gender, socioeconomic status and living in a severely impacted area.

Furthermore, in order to evaluate the impact of the duration of lockdown and of other related containment measures on the primary outcome, the categorical variable “week” was also included in the regression models. The models were adjusted for the rate of new COVID-19 cases and COVID-19-related mortality during the study period, as well as for several socio-demographic characteristics, such as gender, age (managed as categorical variable), occupational status, having a physical illness, levels of perceived loneliness, general health status, taking pharmacological agents for comorbid mental health conditions, coping strategies, and presence of insomnia symptoms. Missing data were handled using the multiple imputation approach. All other variables were managed as previously reported. This statistical approach has been used in previous papers based on COMET data (3).

Statistical analyses were performed using the Statistical Package for Social Sciences (SPSS), version 26.0 and STATA, version 15. For all analyses, the level of statistical significance was set at *p* < 0.005.

## Results

The global sample consisted of 20,720 people. Six percent (*N* = 1,133) of them had pre-existing mental disorders (Table 1). Eighty-two percentage of participants (*N* = 16,899) reported to have frequently searched on Internet information related to the pandemic; they were predominantly female (70.97%, *N* = 11,993), with a mean age of 40.4 ± 0.11 years. Participants reported a significant increase in time spent on Internet since the beginning of the pandemic, with an average time of 4.8 ± 0.02 h per day. No differences among healthcare workers, people infected by COVID-19 and people with pre-existing mental disorders were found in levels of self-reported increased time spent on Internet. However, people with pre-existing mental disorders spent significantly more time on Internet, with 5.2 ± 0.1 h compared to 4.9 ± 0.02 h of the remaining sample (*p* < 0.005).

**Table 1:** Socio-demographic and clinical characteristics of the sample (*N* = 20,720).

Age, years, mean ± SD	40.4 ± 14.3
Age groups, % (*N*)	
18–24 years old	15.2 (3,151)
25–55 years old	65.2 (13,514)
55–64 years old	14.0 (2,904)
Over 65 years old	5.6 (1,151)
Gender, F, % (*N*)	71 (14,720)
Living with partner, yes, % (*N*)	52.2 (10,808)
University degree, yes, % (*N*)	62 (12,844)
Employed, yes, % (*N*)	70 (14,518)
Lost job due to the pandemic, yes, % (*N*)	6.3 (1,302)
Are you practicing smart working, yes, % (*N*)	34.2 (7,089)
Spending more time on Internet, yes, % (*N*)	80.1 (16,598)
Any comorbid physical condition(s), yes, % (*N*)	14.5 (3,012)
Any mental health problem(s), yes, % (*N*)	5.5 (1,133)
Have you been infected by COVID-19, yes, % (*N*)	1.4 (296)
Have you been isolated due to COVID-19 infection, yes, % (*N*)	1.5 (316)
Have you been in contact with someone affected by COVID-19, % (*N*)	4.2 (866)
**Clinical characteristics**	
General health questionnaire—global score, mean ± SD (range: 0–12)	5.6 ± 1.6
Obsessive compulsive inventory—global score, mean ± SD (range: 0–72)	10.7 ± 8.2
Insomnia severity index, mean ± SD (range: 0–28)	9.8 ± 5.2
Suicidal ideation attributes scale (SIDAS), mean ± SD (range: 0–50)	4.9 ± 6.6
Severity of acute stress symptoms—adult, mean ± SD (range: 0–28)	6.0 ± 4.9
**Impact of event scale**, mean ± SD (range: 0–5)	
Intrusion	1.1 ± 1.9
Avoidance	2.3 ± 2.0
Hyperarousal	2.5 ± 1.9
Loneliness, mean ± SD (range: 0–24)	19.1 ± 3.6
**Coping strategies,** mean ± SD (range: 1–4)	
*Maladaptive strategies*	
Self-distraction	2.7 ± 0.8
Denial	1.5 ± 0.7
Venting	2.7 ± 0.8
Behavioural disengagement	1.6 ± 0.6
Self-blame	2.4 ± 0.8
Substance use	1.2 ± 0.5
*Adaptive strategies*	
Acceptance	3.1 ± 0.7
Active	2.9 ± 0.8
Emotional support	2.4 ± 0.8
Use of information	2.4 ± 0.8
Positive reframing	2.3 ± 0.7
Planning	3.0 ± 0.8
*Other*	
Religion	1.9 ± 0.9
Humour	2.1 ± 0.8
**Post-traumatic growth inventory,** mean ± SD (range: 0–10)	
Personal strength	2.1 ± 3.4
Spiritual change	3.7 ± 2.9
Appreciation for life	6.4 ± 3.2
Relating to others	5.3 ± 1.6
New possibilities	5.8 ± 1.6
**Connor—resilience scale**, mean ± SD (range: 0–40)	31.3 ± 10.4
**Multidimensional scale of perceived social support,** mean ± SD (range: 4–28)	
Family support	21.1 ± 6.7
Friends support	20.3 ± 6.5
Support from other relevant ones	22.3 ± 6.7

**Figure 1:**
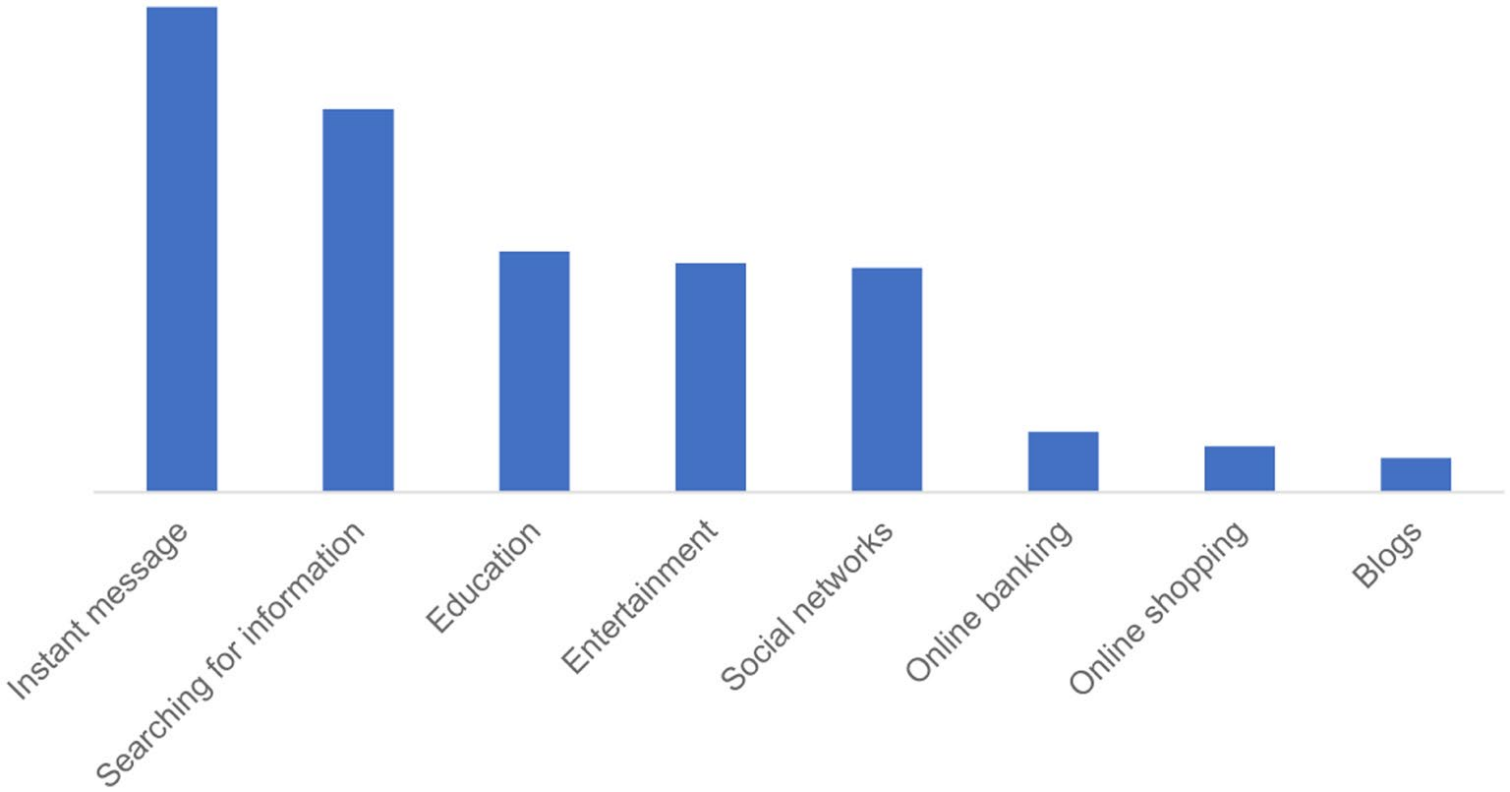
Most frequent reasons for using Internet in the global sample (*N* = 20,720).

The most frequent purposes for accessing Internet were instant messages (35.5%, *N* = 7,357), searching for information (28.1%, *N* = 5,803) and searching for educational contents related to the pandemic (17.6%, *N* = 3,642) (Figure 1). People with pre-existing mental disorders less frequently used Internet for searching information regarding the pandemic compared to the remaining sample (*p* < 0.005), while no significant differences were found in respect to the other purposes for accessing Internet. Moreover, people aged 25–55 years old were those using more frequently Internet for searching information about the pandemic, but such difference was not confirmed in the subgroup of people disclosing to have a pre-existing mental disorder.

**Figure 2:**
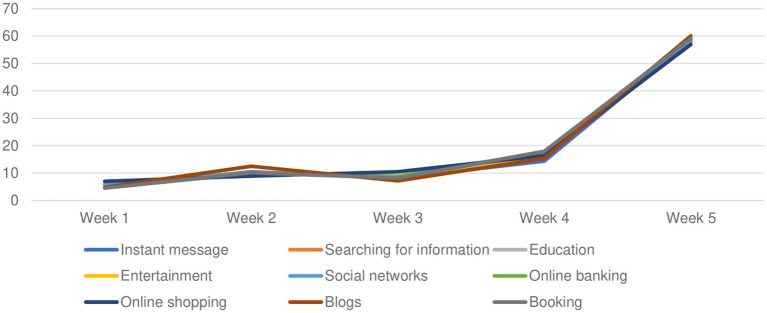
Changes in usage of Internet over lockdown weeks.

A significant increase in usage of social network and for searching for information related to the pandemic was found as long as the pandemic was going on (Figure 2).

The levels of anxiety symptoms were higher in people reporting a more frequent use of social media (7.3 ± 0.1 vs. 7.5 ± 0.05, *p* < 0.005), while no differences were found in levels of depressive and stress symptoms, neither in obsessive-compulsive symptoms, stress-related symptoms, suicidal ideation, recovery style and levels of loneliness.

According to the multivariate logistic regression model, people presenting moderate levels of depressive symptoms (OR: 1.26, CI 95%: 0.99 to 1.59, *p* < 0.005) were at higher risk for an excessive use of social media and Internet. On the contrary, being a student (OR: 0.72, CI 95%: 0.53 to 0.96, *p* < 0.0029) and living in central Italy (OR: 0.46, CI 95%: 0.23 to 0.90, *p* < 0.002) were protective factors. Weeks of lockdown, coping strategies, levels of anxiety and stress symptoms, resilience style and working conditions were not associated with the risk of having an excessive use of social media and Internet (Table 2). Moreover, people with pre-existing mental disorders, those infected by COVID-19 and healthcare professionals did not have a higher risk of excessive use of social media/internet.

**Table 2:** Logistic regression for identifying predictors of using social media.

	***p***	**Sig.**	**Confidence Interval 95%**	
			Lower bound	Upper bound
Intercept	3.71	0.004	1.05	13.08
*Time to exposure, ref. week March 30–April 8*				
Week April 15–April 9	1.37	0.172	0.87	2.16
Week April 16–April 22	1.08	0.745	0.64	1.82
Week April 23–April 29	1.58	0.118	0.88	2.82
Week April 30–May 4	1.28	0.474	0.64	2.56
Quarantine, yes	0.027	0.702	−0.110	0.163
*Geographic area, ref. southern Italy*				
Northern region	0.98	0.858	0.79	1.204
Central region	0.464	0.002	0.23	0.91
Islands	0.94	0.631	0.75	1.18
Gender, female ref.	1.08	0.417	0.89	1.31
Healthcare worker	1.139	0.293	0.86	1.45
Being infected by COVID	0.927	0.695	0.63	1.34
Pre-existing mental disorder	0.864	0.489	0.572	1.305
Pre-existing physical disorder	1.011	0.929	0.78	1.30
*Age group, ref. over 65 years old*				
18–24 years old	0.88	0.758	0.39	1.97
25–55 years old	0.75	0.389	0.39	1.44
55–64 years old	0.70	0.311	0.35	1.38
DASS-stress				
Mild	0.85	0.246	0.65	1.11
Moderate	0.84	0.223	0.64	1.10
Severe	1.15	0.570	0.69	1.92
DASS-anxiety				
Mild	0.94	0.680	0.72	1.23
Moderate	0.92	0.551	0.70	1.20
Severe	1.08	0.319	0.57	1.92
DASS-depression				
Mild	0.95	0.732	0.72	1.24
Moderate	1.26	0.005	0.99	1.59
Severe	1.05	0.724	−0.77	1.23
Resilience level	1.01	0.582	0.99	1.01
Avoidant coping	1.01	0.615	0.96	1.06
Approach coping	0.98	0.357	0.94	1.01
GHQ total	1.01	0.438	0.98	1.03
Insomnia, yes	0.88	0.216	0.72	1.07
Loneliness total score	1.09	0.329	0.91	1.30
Stress symptoms	1.00	0.411	0.98	1.03
Sidas total score	1.01	0.085	0.99	1.02
*Civil_status, divorced*				
Single	0.74	0.104	0.51	1.06
With partner/married	0.81	0.220	0.58	1.13
Widow	1.63	0.375	0.55	4.85
Cases COVID	0.99	0.312	0.99	1.00
Death COVID	1.00	0.292	0.99	1.00
Smart working, yes	0.905	0.325	0.74	1.10
Student, yes	0.72	0.029	0.53	0.96
Lost job, yes	0.76	0.124	0.53	1.07

## Discussion

During the initial stages of the pandemic, many people felt confused and overwhelmed by the excess of information available online. Almost 5 million COVID-19-related messages were disseminated daily (14, 49), causing an “information overload.” Such phenomenon can cause mental distress both in case of inappropriate information as well as in case of an overabundance of correct information. However, the use of social media and of Internet has also been a useful strategy to cope with the unexpected changes in ordinary life caused by the severe restrictive measures issued for containing the pandemic. Therefore, it is relevant to understand the trends in using social media and Internet in Italy, which represents a country with specific cultural values and low levels of digitalization of the general population compared to other countries (50). The most relevant strength of our study is the provision of a snapshot of social media and Internet usage from a vast sample of the Italian general population during the most severe and complicated phase of the COVID-19 pandemic.

In particular, the average time spent on Internet reported by the participants was about 6 h per day, which is in line with the most recent figures from the GlobalWebIndex (GWI) (51). However, this data should be carefully evaluated when considering specific at-risk population, such as adolescents or people with mental disorders, for whom spending almost half of their waking hours on online activities might result in increased mental distress (52). In our survey we found that people with mental disorders spent significantly more time on Internet compared to the general population, which is in line with other studies showing that rates of social media use among people with mental disorders have increased in recent years (53). In particular, it may be that patients with mental disorders use social media and Internet to overcome social isolation and discrimination experienced during *in-vivo* interactions (54). Another possible reason is that people with pre-existing mental disorders—already reporting high levels of anxiety and stress symptoms—more frequently searched information online to overcome uncertainty and fears (55). It would be important to explore the relationship between use of social media and Internet and the levels of personal functioning of people with severe mental disorders. This was not done in our study since we could only assess participants’ self-reported clinical symptoms, coping strategies and resilience styles, but we did not collect any information on personal and social functioning. In the next future, long-term observational studies, including detailed assessments of patients’ social and personal functioning, could clarify the impact of social media and Internet use on the levels of patients’ personal and social functioning.

When considering sample composed by adolescents and/or young adults suffering from psychotic disorders and mood disorders, it has been reported that over 97% uses social media daily (56). Therefore, the risks of using social media should be highlighted. Of course, Internet and social media also can play a positive role for mental health by fostering interactions with others, engaging with peer support networks, and accessing information and services (17, 57), and thus the boundaries for an appropriate, coherent, and potential beneficial usage of Internet should be established (58). To improve the usage of social media, it would be useful to better study and understand the modality of using social media in young people and in their peers with mental health problems (59–62).

Most participants declared to use Internet and social media for instant messaging. Considering the stay-at-home orders issued during the data collection period, this finding is not surprising at all. However, the risk is that people will continue to prefer at-distance to face-to-face interactions, even when the pandemic status is over (63). As pointed out by Osler and Zahavi (64), technology can modify “the embodied experience of the other” and “digital meetings” define new forms of sociality, with specific long-term consequences on mental health.

Internet and social media were used by almost 30% of the sample for searching information about the pandemic. This data can be paired with that of the unprecedented huge volume of information disseminate on the web. Such information are frequently inaccurate or false, nurturing uncertainty and anxiety in those searching information and generating “infodemic” phenomenon. Moreover, the strategy of searching for information in order to control or manage anxiety is often counterproductive, while it usually tends to increase anxiety levels and should even worsen mental health problems (65, 66). In the context of an infodemic—as during the COVID-19 pandemic—people spending an excessive amount of time and energy in over-interpreting information are exposed to a significant risk to negatively react to the stressful situation (10).

The present study has some limitations, which are hereby acknowledged. First, the adoption of the online snowball sampling methodology should have caused a selection bias. In particular, it could be that only people interested in the psychological and psychiatric consequences of the pandemic should have decided to take part to the survey, as well as those more confident in using online tools (67, 68). Secondly, the survey is cross-sectional and therefore it is not possible to define any causal relationship among selected variables. Third, the use of a non-validated tool to get information on Internet use should be acknowledged. Thus, the presence of Internet addiction or pathological social media use could not be assessed. However, the items to evaluate social media and Internet usage were chosen by a group of experts, including the chair of the Section of Digital Psychiatry of the European Psychiatric Association.

## Conclusion

The assessment of social media and Internet usage in the general population during the initial stages of a public health emergency plays a key-role in the development of specific preventive and supportive interventions and for listing suggestions on how to safely use Internet and social media (17). The consequences of the COVID-19 pandemic on global mental health and wellbeing could be longstanding and far-reaching both for the general population, and especially for at-high-risk groups, such as people with pre-existing mental disorders (69–73). Therefore, it is necessary to identify potential risks and protective factors for an excessive use of social media and Internet in order to balance the pros and cons before including web-delivered psychiatric interventions into therapeutic programmes proposed to people with mental disorders (74–77). Lifestyle medicine might represent an optimal strategy to support the adaptation to the post-COVID condition in the general population. In fact, the COVID-19 aftermath represents an opportunity for implementing changes in lifestyle behaviours, since people is more receptive to change (78). Therefore, policy changes promoting lifestyle modifications can be disseminated and integrated into plans to rebuild society. The adoption of a healthier “new normality” is easier in the COVID-19 aftermath. Healthy lifestyle behaviours, which include balanced diet, smoking free and physical exercise, can help the promotion of mental health and the prevention of mental disorders in the general population as well as in specific at-risk population, such as people with severe mental disorders.

## Data availability statement

The raw data supporting the conclusions of this article will be made available by the authors, without undue reservation.

## Ethics statement

The studies involving human participants were reviewed and approved by the Ethical Review Board of the University of Campania “L. Vanvitelli” (protocol number: 0007593/i). The patients/participants provided their written informed consent to participate in this study.

## Author contributions

GiS, MV, ML, BiD, UA, CC, GC, FC, BeD, MN, MP, GbS, AT, UV, and AF have collaborated to the writing and revision of the paper. All authors contributed to the article and approved the submitted version.

## Conflict of interest

The authors declare that the research was conducted in the absence of any commercial or financial relationships that could be construed as a potential conflict of interest.

## Publisher’s note

All claims expressed in this article are solely those of the authors and do not necessarily represent those of their affiliated organizations, or those of the publisher, the editors and the reviewers. Any product that may be evaluated in this article, or claim that may be made by its manufacturer, is not guaranteed or endorsed by the publisher.
