# Supplementary material for: The effect of social media and infodemic on mental health during the COVID-19 pandemic: results from the COMET multicentric trial
Source: Front Psychiatry. 2023 Jul 27;14:1226414. doi: 10.3389/fpsyt.2023.1226414 (PMC10412935; doi:10.3389/fpsyt.2023.1226414)
Supplement: Supplementary file 1 [file Data_Sheet_1.docx]

**Supplementary material 1**

**Figure 1. Survey items on usage of Internet**

- Since the declaration of the pandemic crisis, are you spending more time on Internet?
- Please rate how frequently do you use Internet for each of the following purposes?
- Instant message
- Searching for information
- Education
- Entertainment
- Social networks
- Online banking
- Online shopping
- Blogs
- Booking
- How much time do you spent on Internet (on average)? (please report minutes or hours)
